# The Influence of the Thickness of Compact TiO_2_ Electron Transport Layer on the Performance of Planar CH_3_NH_3_PbI_3_ Perovskite Solar Cells

**DOI:** 10.3390/ma14123295

**Published:** 2021-06-14

**Authors:** Andrzej Sławek, Zbigniew Starowicz, Marek Lipiński

**Affiliations:** 1 Institute of Metallurgy and Materials Science, Polish Academy of Sciences, ul. Reymonta 25, 30-059 Kraków, Poland; aslawek@agh.edu.pl (A.S.); z.starowicz@imim.pl (Z.S.); 2 Academic Centre for Materials and Nanotechnology, AGH University of Science and Technology, al. Mickiewicza 30, 30-059 Kraków, Poland

**Keywords:** titanium dioxide, thin film, perovskite solar cells, electron transport layer

## Abstract

In recent years, lead halide perovskites have attracted considerable attention from the scientific community due to their exceptional properties and fast-growing enhancement for solar energy harvesting efficiency. One of the fundamental aspects of the architecture of perovskite-based solar cells (PSCs) is the electron transport layer (ETL), which also acts as a barrier for holes. In this work, the influence of compact TiO_2_ ETL on the performance of planar heterojunction solar cells based on CH_3_NH_3_PbI_3_ perovskite was investigated. ETLs were deposited on fluorine-doped tin oxide (FTO) substrates from a titanium diisopropoxide bis(acetylacetonate) precursor solution using the spin-coating method with changing precursor concentration and centrifugation speed. It was found that the thickness and continuity of ETLs, investigated between 0 and 124 nm, strongly affect the photovoltaic performance of PSCs, in particular short-circuit current density (J_SC_). Optical and topographic properties of the compact TiO_2_ layers were investigated as well.

## 1. Introduction

**Figure 1:**
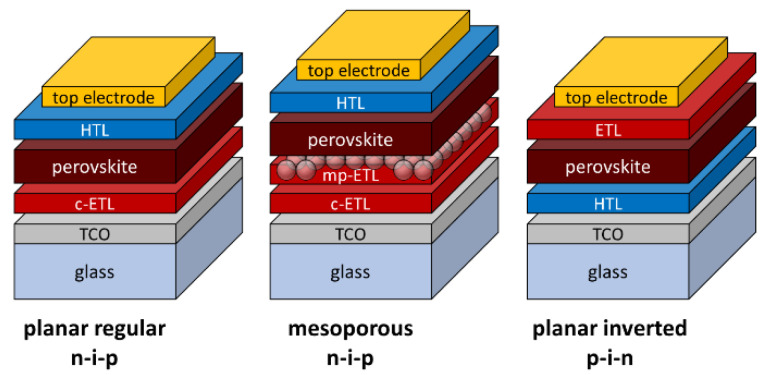
Schemes of the three most common architectures used in PSC technology, from left to right: planar regular (n–i–p), mesoporous (n–i–p) and planar inverted (p–i–n). The devices are composed of glass coated with transparent conductive oxide (TCO), an electron transport layer (ETL), a possibly mesoporous electron transport layer (mp-ETL), the perovskite, a hole transport layer (HTL) and the top electrode.

One of the most reasonable responses for growing global electricity demands, respecting the need to protect the natural environment, is harnessing solar energy by means of photovoltaic devices. Besides the commonly used mature wafer-based silicon technology, the new branch of perovskite-based solar cells is very promising due to the high performance and potentially low costs of production. Since the first report in 2009 by Miyasaka’s group [1], the efficiency has advanced from 3.8% to more than 25% [2]. Perovskite solar cell (PSC) technology evolved at first from titania-based dye-sensitized solar cells [3] and spread later into different architectures [4]: regular n–i–p with and without a mesoporous TiO_2_ layer or inverted p–i–n (Figure 1). Although slightly higher efficiencies are obtained with mesoporous titania scaffolds [5], planar structures without them are more prospective from an industrial point of view. After it was proved that perovskites, unlike organic absorbers, generally have a long carrier diffusion length (a few hundreds of nanometers) [6], mesoporous TiO_2_ was able to be omitted, allowing for the processing of whole solar cells below 200 °C. High efficiency of 21.6% for the planar n–i–p structure has been reported by Jiang et al. [7], while other authors have claimed an even higher value of 23.7% [8]. In general, the PSC structure is composed of a perovskite absorber layer placed between a selective transport layer for holes (HTL) and electrons (ETL). For the p–i–n structure, the most commonly used HTLs are PEDOT:PSS [9] and NiO_x_ [10], while the ETL, mostly PCBM [11], is placed on top of the HTL and perovskite. On the other hand, in the most common n–i–p architecture, titanium dioxide is predominantly used as an ETL followed by a perovskite absorber and Spiro-OMeTAD as the HTL. Although significant progress in PSCs was enabled by engineering the perovskite composition [12,13] and microstructures [14,15], achieving high power conversion efficiency is not possible without optimizing other cell components.

The ETL in PSCs transports electrons generated from the perovskite layer [16]. It also serves as a blocking layer to hinder direct contact between the holes and FTO [17]. Hence, the layer should be pinhole free to prevent the recombination of electrons and holes at the front electrodes. Compatible energy levels support the fast injection of photogenerated electrons from perovskites and low-voltage losses. It also acts as a window layer for the perovskites; thus, a bandgap above 3 eV is necessary. The layer should electrically be as thin as possible to provide fast electron transport and low resistive losses. The titanium-oxide-based ETL is also a crucial investigation area reported in a number of studies [18,19,20,21,22]. The titanium dioxide blocking layer can be deposited by various methods: atomic layer deposition (ALD) [23], magnetron sputtering [24], spray pyrolysis [25], electrochemical deposition [26] and spin coating, where the latter is most commonly used. Due to the relatively high surface roughness of the FTO electrode, typically ca. 20 nm (Rq), the deposition methods can be divided into methods supporting a perfectly conformal morphology, such as ALD or sputtering, and methods resulting in fewer conformal layers such as spin coating. Spin-coated compact TiO_2_ layers more easily fill the troughs of the rough surface than the peaks. The optimal thickness of c-TiO_2_ is a minimal one that supports the blocking properties at the peaks. Literature reports have announced a variety of titanium precursors and deposition conditions; however, the optimal thickness is rather rarely given explicitly. Additionally, each precursor may require different thicknesses to reach the highest cell performances, as discussed in [27], the reason being that there are intrinsic differences in material properties in comparison to the morphological aspects. For instance, Qin et al. compared three different titanium precursors: tetrabutyl titanate, titanium diisopropoxide bis(acetylacetonate), titanium isopropoxide and solvents [28], concluding the first as the best. However, the thicknesses of compared layers differed considerably. Furthermore, the confrontation of ETLs was conducted in the presence of mesoporous TiO_2_, which could slightly disrupt the thickness effects, and the surface roughness of FTO used in these studies was approximately two times lower than commonly used.

It is known that ETLs (TiO_2_ or SnO_2_) should be uniform, pinhole free and completely cover the surface of FTO. Therefore, the optimal thickness depends on the deposition technique and post-treatment of these layers. Many authors have reported thin ETLs in the range of 20–30 nm. For example, Xiao et al. [29] applied a 30 nm thick layer of TiO_2_ for the planar n–i–p structure and obtained a power conversion efficiency of 13.9%. Saliba et al. [4] used 20–30 nm thick SnO_2_ (instead of TiO_2_) in the planar regular structure (n–i–p) and a 20–30 nm compact TiO_2_ layer for the mesostructure for high-efficiency solar cells with a PCE ≥20%. On the other hand, Sun et al. [30] obtained the greatest efficiency (PCE of 18.32%) for much thicker layers of 152 nm. In this case, the TiO_2_ layer was additionally treated with TiCl_4_. However, for the ALD layer deposition technique, the thickness of the layers could be much lower. Lu et al. [31] showed that the optimal thickness for the ALD technique is considerably smaller and equal to 10 nm (PCE of 13.6%). This suggests that the ETL may be very thin but must also be of good quality.

Considering the ETL is an important part of PSCs, in this work, we emphasize its impact on perovskite solar cell parameters. The regular planar n–i–p architecture of PCSs was studied, i.e., glass/FTO/c-TiO_2_/MAPbI_3_/Spiro-OMeTAD/Au. In this report, we deposited ETLs using a titanium diisopropoxide bis(acetylacetonate) (Ti(acac)_2_OiPr_2_) precursor solution in 1-BuOH. This set was chosen because its good performance matches the preparation simplicity and good storage stability. This stability is important in the case of experimental repeatability, as it was already reported for precursor solutions undergoing aging processes [27,32]. Thoroughly differing thicknesses of the compact TiO_2_ layer were the key features enabling careful investigation of the properties of c-TiO_2_ and their influence on the performance of PSCs. Complex studies enabled a better understanding of the observed effects.

## 2. Materials and Methods

All reagents were used without purification: titanium diisopropoxide bis(acetylacetonate) (Ti(acac)_2_OiPr_2_) (75 wt % in isopropanol, Sigma-Aldrich); 1-butanol (1-BuOH) (99.5%, Chempur); chlorobenzene (CB) (99.5%, Chempur); acetonitrile (99.8%, Sigma Aldrich); PbI_2_ (99.99%, TCI); methylammonium iodide (MAI) (>99.99%, Greatcell Solar Materials); γ-butyrolactone (GBL) (≥99%, Sigma-Aldrich, St. Louis, MO, USA); dimethyl sulfoxide (DMSO) (99.9%, Sigma-Aldrich, St. Louis, MO, USA); N^2^,N^2^,N^2^′,N^2^′,N^7^,N^7^,N^7^′,N^7^′-octakis(4-methoxyphenyl)-9,9′-spirobi [9H-fluorene]-2,2′,7,7′-tetramine (Spiro-OMeTAD) (99%, Sigma-Aldrich, St. Louis, MO, USA); 4-tert-butylpyridine (TBP) (98%, Sigma Aldrich); bis(trifluoromethane)sulfonimide lithium salt (LiTFSI) (99%, Sigma-Aldrich, St. Louis, MO, USA).

### 2.1. Preparation of Compact TiO_2_ Layers

For the electron transport layer (ETM), we used compact TiO_2_ (c-TiO_2_) layers of different thicknesses. They were deposited either on square (2 cm × 2 cm) FTO glass substrates (Sigma-Aldrich, St. Louis, MO, USA, ~7 Ω/sq) or on a (100) polished silicon wafer. FTO glass plates were washed by dipping in hot 2% (*v/v*) Hellmanex^®^ III (Hellma Analytics, Müllheim, Germany) solution in deionized (DI) H_2_O in an ultrasonic cleaner for 5 min. After that, they were dipped in hot DI H_2_O and again in isopropanol in an ultrasonic cleaner for 5 min. Finally, the plates were submerged in DI H_2_O and dried. Silicon wafers were cleaned successively with acetone and isopropanol and rinsed with deionized water. Just before applying compact TiO_2_ layers, FTO glass plates and Si wafers were additionally treated with O_2_ plasma (Harrick Plasma, Ithaca, NY, USA) for 15 min (MID).

The c-TiO_2_ precursors were prepared by mixing 75 wt % Ti(acac)_2_OiPr_2_ in isopropanol (IPA) with 1-BuOH. Zero, 0.243, 0.486, 0.729, 1.214, 1.821 and 2.428 g of 75 wt % Ti(acac)_2_OiPr_2_ was added to a volumetric flask and filled to 5 mL with 1-BuOH to make a 0, 0.1, 0.2, 0.3, 0.5, 0.75 and 1 M (mole/dm^3^) solution. A 70 µL volume of precursor solution was spread on cleaned FTO/Si plates and spin coated at 2000, 3000 or 4000 revolutions per minute (RPM) for 15 s. They were then preheated at 200 °C for 10 min in an oven and calcined at 500 °C for 30 min in a tube furnace in the presence of air.

### 2.2. Fabrication of Solar Cells

Deposition of lead iodide (MAPbI_3_) and the hole transport layer (HTL) was performed in a nitrogen-filled glove box (MBRAUN, Garching, Germany). A ca. 1 M methylammonium MAPbI_3_ precursor was prepared by dissolving 0.461 g (1 mmol) of PbI_2_ and 0.159 g (1 mmol) of MAI in 700 µL of GBL and 300 µL of DMSO at room temperature (RT) at ca. 28–30 °C. The solution was stirred with a magnetic stirrer for ca. 3 h and then filtered through a syringe filter of a 0.45 μm membrane pore size. Prior to deposition of the precursor, FTO/c-TiO_2_ substrates were cleaned in O_2_ plasma for 30 min (MID). A 70 µL volume of the MAPbI_3_ precursor was spread on the FTO/c-TiO_2_ plate by two-step spin coating: 1000 RPM (200 RPM/s ramp) for 10 s and immediately 5000 RPM (1333 RPM/s ramp) for 20 s. Between 16 and 18 s of 5000 RPM spinning, 200 µL of toluene antisolvent was added. The spin-coating program is presented in Figure S1 of the Supporting Information. After deposition of MAPbI_3_, each sample was annealed at 100 °C on a hot plate for 10 min and cooled down to RT.

To deposit the hole transport layer (HTL), we used a stock solution of doped Spiro-OMeTAD in CB. A 0.368 g (0.3 mol) amount of Spiro-OMeTAD was dissolved in 5 mL of CB and doped with 148 μL (1.01 mol) of TBP and 83 μL of LiTFSI (0.15 mol) stock solution (520 mg/mL in acetonitrile). A 70 µL volume of the Spiro-OMeTAD solution was spread on the MAPbI_3_ perovskite layer and spin coated at 4000 RPM for 30 s. After that, the samples were taken out of the glove box, masked and coated with gold by thermal evaporation. Au electrodes had a surface of 0.25 cm^2^ and a thickness of approximately 120 nm.

### 2.3. Apparatus Used in Measurements

Photovoltaic performance measurements were carried out by I–V curve tracing using a Photo Emission Tech AAA class solar simulator under standard test conditions. The microstructural and surface investigations were performed using Innova multimode atomic force microscopy and scanning electron microscopy (tabletop TM3030, Hitachi High-Tech, Tokio, Japan). The optical characterization was based on ellipsometry (SE800 PV, SENTECH Instruments, Berlin, Germany, range = 300–980 nm, incident angle = 70°) and UV–vis–NIR spectroscopy (Lambda 950S, Perkin Elmer, Waltham, MA, USA, range = 300–850 nm).

## 3. Results and Discussion

### 3.1. Fabrication of c-TiO_2_ Thin Layers by Spin Coating

**Figure 2:**
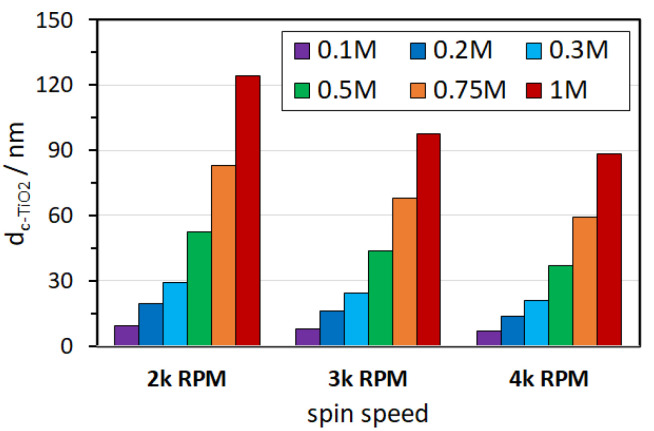
The dependence of the thickness of the c-TiO_2_ layer on the spin speed (in 10^3^ revolutions per minute) in the spin-coating process for different concentrations of Ti(acac)_2_OiPr_2_ in the 1-BuOH precursor.

Spin coating is a common technique used for the deposition of thin films on solid substrates. The main advantage of this process is the ability to quickly and easily produce very fine and uniform coatings in the thickness range of micrometer to nanometer [33]. The thickness of the layer depends on many factors and can be described by the model of Mayerhofer [34]:

(1) $$d=\left(1-\frac{\rho_{0}}{\rho }\right)\cdot {\left(\frac{3\eta \cdot m}{2\rho_{0}\omega^{2}}\right)}^{\frac{1}{3}}$$

where *d* is the thickness, *ρ* is the density of the solution, *ρ*_0_ is the density of the solvent, *η* is the viscosity of the solution, *m* is the rate of evaporation and *ω* is the angular speed. In practice, the thickness of films deposited from solutions is controlled by the spinning speed (related to angular velocity) and concentration of the desired substance (related to viscosity). In this work, we coated glass/FTO substrates using a solution of titanium diisopropoxide bis(acetylacetonate) (Ti(acac)_2_OiPr_2_) in 1-butanol (1-BuOH) and annealed it at 200 and 500 °C. A commercially available 75% solution of Ti(acac)_2_OiPr_2_ in isopropanol mixes with 1-BuOH in any ratio, forming clear mixtures that do not age quickly (no rapid hydrolysis process). In this work, we investigate a series of 19 devices with a thickness of c-TiO_2_ layers ranging from 0 to 124 nm, which were deposited by the spin-coating method with varying centrifugation speed and concentration of the precursor. The layer thickness was associated with the nominal thickness of polished Si determined from ellipsometry. Figure 2 shows the dependence of the thickness of the c-TiO_2_ layer on the centrifugation speed for solutions of different concentrations. It can be seen that the precursor concentration has a major influence on the thickness of the c-TiO_2_ film. Generally, the c-TiO_2_ layer formed at 2000 RPM can be thinned by 13–21% using 3000 RPM or by 26–31% using 4000 RPM. The full set of ellipsometry data is available in Table S1 in the Supporting Information.

Figure 1 shows schemes of the three most common architectures of PCSs: planar regular (n-i-p), mesoporous (n-i-p) and planar inverted (p-i-n) [35]. Because this publication focuses on the compact (or blocking) TiO_2_ layer, we used the simple planar regular (n-i-p) architecture of the glass/FTO/c-TiO_2_/MAPbI_3_/Spiro-OMeTAD/Au configuration with no mesoporous scaffold. Herein, for the most part, the light reaches the perovskite absorber through the glass substrate, FTO and electron transport layer (ETL), c-TiO_2_. Light nonabsorbed during the first pass (λ > 600 nm) is mostly backscattered from the gold electrode, as it results from its complex refractive index [36].

### 3.2. Optical Absorption

**Figure 3:**
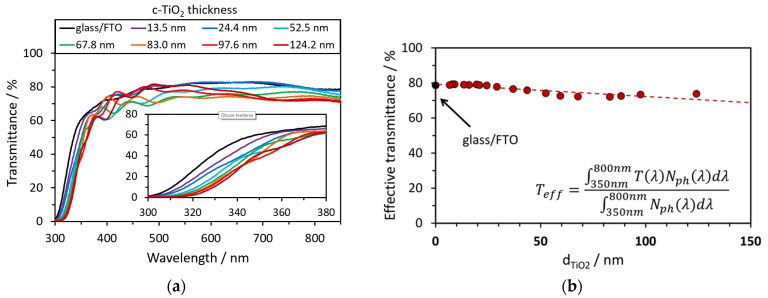
(**a**) Transmittance of glass/FTO/c-TiO_2_ of different thicknesses obtained from the Ti(acac)_2_OiPr_2_ solution in 1-BuOH; (**b**) the dependence of the effective transmittance on the thickness in glass/FTO/c-TiO_2_. Effective transmittance T_eff_ was calculated for the range of wavelengths λ from 350 to 800 nm, where T is the transmittance, and N_ph_ is the radiant flux.

The power conversion efficiency (PCE) of PSC strongly depends on the photocurrent, which, in turn, is directly proportional to the number of absorbed photons. It is well known that MAPbI_3_ perovskite is an excellent absorber in the 300–800 nm wavelength range [37]. The onset photon absorption energy of polar MAPbI_3_ perovskite is about 1.5 eV (≈827 nm), which is close to its electronic bandgap value of 1.55 eV (≈800 nm) [38]. It shows a higher quantum yield in the blue/green region and a lower yield in the red/infrared [39,40]. For this reason, the ETL material should be as transparent as possible in a wide range of spectra. The thickness of the c-TiO_2_ layer clearly affects the optical transmission of perovskite, which is shown in Figure 3. The used glass/FTO substrate absorbs or reflects some of the light, ca. 20% between 450 and 850 nm, and much more in the near-ultraviolet range (300–350 nm). c-TiO_2_ layers less than ca. 30 nm hardly affect the transmittance of glass/FTO, except for some absorption below ca. 380 nm, which is characteristic of anatase [41]. The effective transmittance of the samples slowly decreases as the thickness of the c-TiO_2_ layer increases (Figure 3b). It is worth noting that for all samples, one can observe multiple maxima and minima above the absorption edge in the transmittance spectra (ca. >360 nm), which are especially pronounced for c-TiO_2_ layers thicker than 24.4 nm. This is due to the interference effect resulting from the two interface boundaries, c-TiO_2_/FTO and the FTO/glass substrate, which suggests that the surface and interface of these bilayers are rather optically smooth [42]. The transmittance data around the absorption band edge follow the clear trend of edge red shift with TiO_2_ layer thickness. Thus, taking into account the bandgap of the anatase TiO_2_ layer on FTO, it should be attributed to parasitic light absorption in TiO_2_. UV–vis–NIR spectra for all the studied samples are available in the Supporting Information (Figure S2).

### 3.3. Atomic Force Microscopy

**Figure 4:**
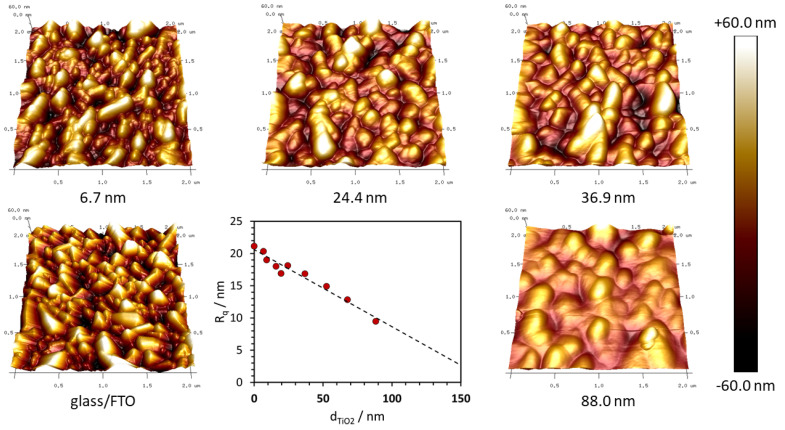
The dependence of the root mean square surface roughness (Rq) on the thickness of the c-TiO_2_ layer deposited on the glass/FTO substrate. It was collated with the selected AFM images of the surface roughness and morphology of the glass/FTO/c-TiO_2_ substrate, where the sampling area was 2 µm × 2 µm. The color scale for height is also shown.

Figure 4 shows that the used glass/FTO substrate exhibits a roughness of ca. 21 nm that decreases linearly with the increasing thickness of c-TiO_2_, down to ca. 9.5 nm for the 88.3 nm thick layer. The topography of the FTO surface shows sharp tops and mild depressions resulting from the columnar grain structure of this material. Based on the AFM profiles, it should be emphasized that even thin c-TiO_2_ layers, e.g., 6.7 nm, dull the sharp peaks effectively, while the thicker layers smooth the entire surface. Due to the right viscosity of the 1-butanol-based titanium diisopropoxide bis(acetylacetonate) sol, the layer shows more conformal growth than other sols such as ethanol-based titanium ethoxide [27]. So far, there is a significant divergence of optimal thickness according to the deposition method: ~10–15 nm for ALD and 50–60 nm for spin coating. The enhanced conformality is the key feature that allows the thinning of the blocking layer. Although the roughness of TiO_2_ could also affect the crystallization of MAPbI_3_, SEM images show a similar microstructure of perovskite films deposited on the substrates with different c-TiO_2_ thicknesses (Figure S3).

### 3.4. Current–Voltage (I-V) Characteristics of Solar Cells

**Figure 5:**
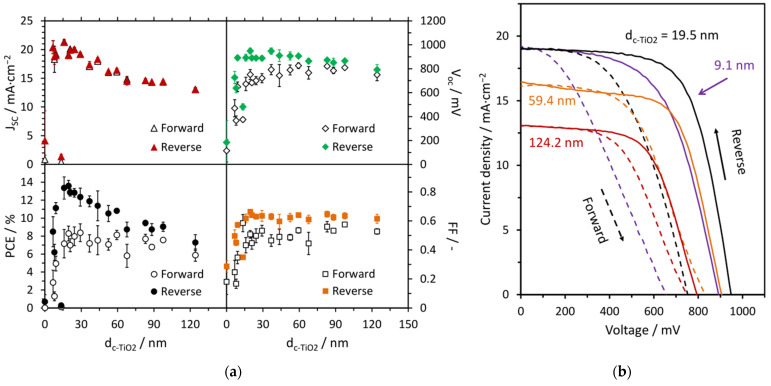
(**a**) Influence of the c-TiO_2_ layer thickness on basic photoelectric parameters: short-circuit current density (J_SC_), open-circuit voltage (V_OC_), power conversion efficiency (PCE) and fill factor (FF). (**b**) Averaged current density–voltage (J–V) curves for the selected cells with different c-TiO_2_ thicknesses (scan speed = 1V/s, light soaking 10 s before measurement).

The electrical properties of solar cells have a direct impact on their performance. Figure 5a shows that the introduction of an even, very thin layer of c-TiO_2_ into the cells results in a step increase of all basic photoelectric parameters compared to the device where perovskite was deposited directly on FTO. However, TiO_2_ electron transport layers below ca. 15 nm are too thin, which lowers the cells’ efficiencies. Additionally, part of such cells turned out to be faulty, and the devices were difficult to repeat. Most likely, such c-TiO_2_ layers may not be completely continuous, and the pinholes present result in the fast electron–hole pair recombination at the FTO/MAPbI_3_ interface. This can also provide an alternate current path for the light-generated current, lowering the Shunt resistance of the cell. The optimal ETL thickness for the studied perovskite-based solar cells was 19.5 nm, resulting in the highest power conversion efficiency (PCE) of 13.6%. Along with thickening of the c-TiO_2_ layer, the fill factor (FF) remains at a similar level (0.60–0.65), the open-circuit voltage (V_OC_) slightly decreases from ca. 950 mV to ca. 800 mV, while the short-circuit current density (J_SC_) drops significantly from 19.0 to 13.1 mA·cm^−2^. This drop cannot be explained only by the lower effective transmittance (Figure 3b), which indicates the hindered transport properties. It is worth noting that the highest J_SC_ of 21.3 mA·cm^−2^ was recorded for the cells with a 15.9 nm c-TiO_2_ layer; however, the remaining parameters (FF and V_OC_) were significantly lower than the cells with the optimal c-TiO_2_ layer. As a result, we observe a gradual decrease in PCE from 13.6% for the 19.5 nm c-TiO_2_ layer to 7.3% for the 124.2 nm layer. The selected electrical parameters are collected in Table 1, while the remaining are available in the Supporting Information (Table S2).

A few selected J–V curves for the studied cells are presented in Figure 5b. They directly reflect the trends observed for the electrical parameters (Figure 5a, Table 1). The shapes of the J–V curves are similar for all cells, which is reflected by similar values of FF. All devices exhibit hysteresis typical for perovskite solar cells between forward and reverse scans, the origins of which are due to ferroelectric polarization, ion migration, charge trapping and/or capacitive effects [43]. In the case of planar heterojunction PSCs under study, the hysteresis is much more pronounced for those with a thinner ETL. Although cells with a c-TiO_2_ layer of 9.1 and 19.5 nm show a similarly high J_SC_, a significantly lower V_OC_ is observed for the thinner layer. It is also clear that the V_OC_ decreases as the c-TiO_2_ thickens.

There may be several reasons for the trends in the electrical properties of the studied PSCs. The thick ETL acts as an insulator for the electrons generated in the absorber, increasing the series resistance of the cell. This hinders charge collection by lowering the gradient of the carrier concentration (diffusion-based transport) and also by disturbing the electric field in the material (drift-based transport).

## 4. Conclusions

The presented results clearly shows that the thickness of the compact TiO_2_ ETL clearly affects the performance of MAPbI_3_ perovskite solar cells. We chose the simplest planar heterojunction architecture (glass/FTO/b-TiO_2_/MAPbI_3_/Spiro-OMeTAD/Au) to minimize the influence of factors that could interfere with the results, e.g., the presence of a mesoporous TiO_2_ scaffold.

The ETL can significantly reduce the roughness of the FTO substrates. Even very thin c-TiO_2_ layers (6.7 nm) dull the sharp grains of FTO, while the thicker layers smooth the entire surface. Compact TiO_2_ also affects the optical transparency of the devices. The effective transmittance of glass/FTO substrates slowly decreases with the thickening of the ETL, but layers thinner than ca. 30 nm still hardly affected it.

More significant effects were observed for the current density–voltage characteristics of the cells. We found that c-TiO_2_ of ca. 20 nm is optimal, providing the best performance of the devices. Thinner layers may result in worse efficiency of cells or difficulties with the reproducibility of the devices. An ETL that is too thin may not be completely continuous and contain pinholes, which may result in fast electron–hole pair recombination at the FTO/MAPbI_3_ interface. It can also provide an alternate current path for the light-generated current, lowering the Shunt resistance of the cell. It is worth noting that even the very thin layer of c-TiO_2_ markedly increases all basic photoelectric parameters compared to the device with perovskite deposited directly on FTO. On the other hand, a TiO_2_ layer that is too thick also has a negative impact on the performance of PSCs, as it significantly reduces the short-circuit current density (J_SC_) and also the open-circuit voltage (V_OC_). Along with the thickening of the c-TiO_2_ layer from ca. 20 nm to ca. 125 nm, J_SC_ decreased by 38% (from 21.3 to 13.1 mA·cm^−2^) and V_OC_ by 16% (from 950 mV to 800 mV). Yet, for most cells, the FF remained at a similar level, between 0.6 and 0.65. In effect, we noticed a clear, gradual decrease in power conversion efficiency (PCE) from 13.6% to 7.3% along with the thickening of the c-TiO_2_ ETL.

## Figures and Tables

**Table 1:** Average electrical parameters and their standard deviations for MAPbI_3_ perovskite solar cells with a compact TiO_2_ layer of different thicknesses (forward scan). The samples are named after the TTDB molar concentration and centrifugation speed.

Sample	d_c-TiO2_ (nm)	J_SC_ (mA·cm^−2^)	V_OC_ (mV)	FF (−)	PCE (%)
Glass/FTO	−	4.2 ± 5.2	188 ± 5.16	0.29 ± 0.04	0.69 ± 0.80
0.1M_2kRPM	9.1	19.1 ± 0.3	891 ± 20	0.57 ± 0.02	11.12 ± 0.62
0.2M_3kRPM	15.9	21.3 ± 0.4	892 ± 28	0.62 ± 0.03	13.36 ± 1.23
0.2M_2kRPM	19.5	19.0 ± 0.2	949 ± 23	0.66 ± 0.02	13.59 ± 0.63
0.5M_4kRPM	36.9	17.4 ± 0.5	947 ± 26	0.63 ± 0.02	11.87 ± 0.61
0.75M_4kRPM	59.4	16.4 ± 0.3	906 ± 11	0.64 ± 0.01	10.81 ± 0.22
1M_2kRPM	124.2	13.1 ± 0.3	791 ± 43	0.62 ± 0.03	7.29 ± 0.88

## Data Availability

Data is contained within the article or supplementary material.

## Supplementary Materials

The following are available online at https://www.mdpi.com/article/10.3390/ma14123295/s1, Table S1: Parameters from ellipsometry measurements for the studied c-TiO_2_ layers on polished silicon wafers, Figure S1: Spin-coating program for the deposition of the MAPbI_3_ perovskite precursor, Figure S2: UV–vis–NIR spectra of the glass/FTO/c-TiO_2_ samples under study, Table S2: Electrical parameters of the studied FTO/b-TiO2/MAPbI_3_/Spiro-OMeTAD/Au solar cells: short-circuit current density (J_SC_), open-circuit voltage (V_OC_), fill factor (FF) and power conversion efficiency (PCE), Figure S3: SEM images showing the morphology of the MAPbI_3_ perovskite layer deposited on glass/FTO/c-TiO_2_.
