# Supplementary material for: The Influence of the Thickness of Compact TiO2 Electron Transport Layer on the Performance of Planar CH3NH3PbI3 Perovskite Solar Cells
Source: Materials (Basel). 2021 Jun 14;14(12):3295. doi: 10.3390/ma14123295 (PMC8232335; doi:10.3390/ma14123295)
Supplement: Supplementary file 1 [file materials-14-03295-s001.zip › materials-1249589-supplementary.pdf]

materials

Supplementary Materials

The Influence of the Thickness of Compact TiO_2 Electron Transport Layer on the Performance of Planar $CH_3NH_3PbI_3$ Perovskite Solar Cells

Andrzej Sławek [1,2], Zbigniew Starowicz [1] and Marek Lipiński [1,*]

[1] Institute of Metallurgy and Materials Science, Polish Academy of Sciences, ul. Reymonta 25, 30-059 Kraków, Poland; aslawek@agh.edu.pl (A.S.); z.starowicz@imim.pl (Z.S.)
[2] Academic Centre for Materials and Nanotechnology, AGH University of Science and Technology, al. Mickiewicza 30, 30-059 Kraków, Poland
* Correspondence: m.lipinski@imim.pl

Citation: Sławek, A.; Starowicz, Z.; Lipiński, M. The Influence of the Thickness of Compact TiO_2 Electron Transport Layer on the Performance of Planar $CH_3NH_3PbI_3$ Perovskite Solar Cells. *Materials* **2021**, *14*, 3295. https://doi.org/10.3390/ma14123295

Academic Editor: Gregory J. Wilson

Received: 21 May 2021
Accepted: 9 June 2021
Published: 14 June 2021

Publisher's Note: MDPI stays neutral with regard to jurisdictional claims in published maps and institutional affiliations.

Copyright: © 2021 by the authors. Submitted for possible open access publication under the terms and conditions of the Creative Commons Attribution (CC BY) license (http://creativecommons.org/licenses/by/4.0/).

Table S1. Parameters from ellipsometry measurements for the studied c-TiO_2 layers on polished silicon wafers. Real part of the refractive index $n_{632.8nm}$ and Mean Square Error indicate fit quality. Final thickness $d_{effTiO2}$ is as sum of thickness of TiO_2 d_{TiO2} and half of roughness d_R.

Sample	$n_{632.8nm}$	MSE	d_{TiO2} [nm]	d_R [nm]	$d_{effTiO2}$[nm]
0.1M_4kRPM	1.9239	0.614	6.7	1.4	6.7
0.1M_3kRPM	1.9027	0.572	7.9	2.2	7.9
0.1M_2kRPM	2.0112	0.499	9.1	4.5	9.1
0.2M_4kRPM	2.007	0.69	13.5	0.2	13.5
0.2M_3kRPM	1.9956	0.591	15.9	0.5	15.9
0.2M_2kRPM	2.0442	0.617	19.5	2.0	19.5
0.3M_4kRPM	2.1271	1.314	20.9	1.0	20.9
0.3M_3kRPM	2.0553	1.276	24.4	1.3	24.4
0.3M_2kRPM	2.0626	1.202	29.2	1.3	29.2
0.5M_4kRPM	2.0734	3.075	36.9	1.6	36.9
0.5M_3kRPM	2.0494	1.472	43.6	0.0	43.6
0.5M_2kRPM	2.1027	3.055	52.5	1.4	52.5
0.75M_4kRPM	2.0622	2.007	59.4	0.0	59.4
0.75M_3kRPM	2.1032	2.811	67.8	2.0	67.8
0.75M_2kRPM	2.0996	4.678	83.0	0.0	83.0
1M_4kRPM	2.0544	2.578	88.3	0.0	88.3
1M_3kRPM	2.1420	9.831	97.6	0.0	97.6
1M_2kRPM	2.1417	7.609	124.2	0.00	124.2

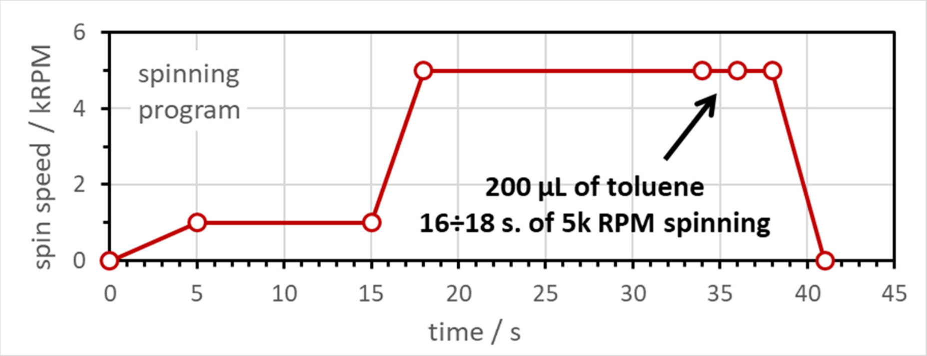

Figure S1. Spin coating program for deposition of $MAPbI_3$ perovskite precursor. Approximate moment of adding toluene antisolvent is marked.

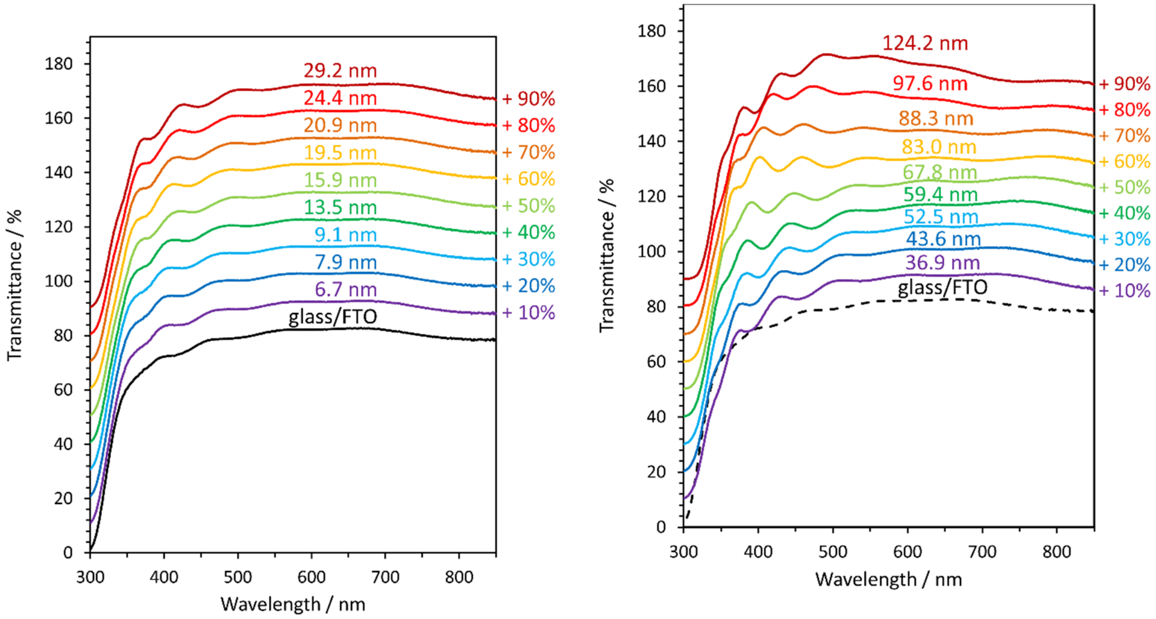

Figure S2. UV-VIS-NIR spectra of the glass/FTO/c-TiO_2 samples under this study. For clarity, the spectra were artificially shifted on the ordinate axis.

Table S2. Electrical parameters of the studied FTO/b-TiO_2/$MAPbI_3$/Spiro-OMeTAD/Au solar cells: short circuit current density J_{SC}, open circuit voltage V_{OC}, fill factor FF and power conversion efficiency PCE. All parameters were averaged for a maximum of 4 separate cells from the same glass substrate, where faulty cells were rejected. The scan rate was 1000 mV·s^{-1} for both forward and reverse scanning directions. Prior to measurements cells were conditioned under Xe lamp for 5 seconds.

Sample	Scan	$d_{c\text{-}TiO2}$ [nm]	No. of cells	J_{SC} [mA/cm^2]	V_{OC} [mV]	FF [–]	PCE [%]
glass/FTO	Reverse	0	4	4.17 ± 5.16	184 ± 184	0.29 ± 0.04	0.68 ± 0.80
	Forward			0.88 ± 0.75	115 ± 94	0.18 ± 0.09	0.02 ± 0.03
0.1M_4kRPM	Reverse	6.7	3	20.38 ± 1.12	726 ± 51	0.50 ± 0.04	8.49 ± 1.68
	Forward			20.41 ± 1.15	470 ± 71	0.25 ± 0.08	2.84 ± 1.37
0.1M_3kRPM	Reverse	7.9	3	18.82 ± 1.41	638 ± 38	0.45 ± 0.02	6.19 ± 0.90
	Forward			18.30 ± 2.28	367 ± 39	0.17 ± 0.03	1.30 ± 0.49
0.1M_2kRPM	Reverse	9.1	4	19.05 ± 0.25	891 ± 20	0.57 ± 0.02	11.12 ± 0.62
	Forward			19.21 ± 0.31	649 ± 40	0.35 ± 0.04	4.95 ± 0.82

0.2M_4kRPM	Reverse	13.5	0	–	–	–	–
	Forward			–	–	–	–
0.2M_3kRPM	Reverse	15.9	4	21.29 ± 0.36	892 ± 28	0.62 ± 0.03	13.36 ± 1.23
	Forward			21.36 ± 0.36	673 ± 56	0.43 ± 0.05	7.16 ± 1.58
0.2M_2kRPM	Reverse	19.5	4	19.01 ± 0.20	949 ± 23	0.66 ± 0.02	13.59 ± 0.63
	Forward			19.07 ± 0.17	751 ± 41	0.51 ± 0.03	8.29 ± 0.83
0.3M_4kRPM	Reverse	20.9	3	19.93 ± 0.05	890 ± 18	0.64 ± 0.01	12.86 ± 0.40
	Forward			20.12 ± 0.12	683 ± 14	0.45 ± 0.04	6.99 ± 0.72
0.3M_3kRPM	Reverse	24.4	4	20.05 ± 0.29	889 ± 14	0.63 ± 0.02	12.82 ± 0.48
	Forward			20.07 ± 0.28	700 ± 34	0.50 ± 0.05	7.98 ± 1.05
0.3M_2kRPM	Reverse	29.2	4	19.21 ± 0.23	887 ± 20	0.64 ± 0.04	12.34 ± 1.00
	Forward			19.23 ± 0.24	721 ± 38	0.53 ± 0.04	8.38 ± 0.99
0.5M_4kRPM	Reverse	36.9	4	17.40 ± 0.53	947 ± 26	0.63 ± 0.02	11.87 ± 0.61
	Forward			17.07 ± 0.55	792 ± 43	0.47 ± 0.04	7.19 ± 0.96
0.5M_3kRPM	Reverse	43.6	3	18.36 ± 0.21	910 ± 49	0.60 ± 0.05	11.38 ± 1.66
	Forward			17.98 ± 0.09	742 ± 92	0.49 ± 0.05	7.54 ± 1.62
0.5M_2kRPM	Reverse	52.5	3	16.24 ± 0.10	907 ± 34	0.62 ± 0.03	10.52 ± 0.90
	Forward			16.02 ± 0.12	790 ± 42	0.49 ± 0.02	7.07 ± 0.63
0.75M_4kRPM	Reverse	59.4	4	16.44 ± 0.26	906 ± 11	0.64 ± 0.01	10.81 ± 0.22
	Forward			16.11 ± 0.22	825 ± 23	0.54 ± 0.02	8.13 ± 0.52
0.75M_3kRPM	Reverse	67.8	4	14.56	864	0.61	8.74

				± 0.76	± 21	± 0.03	± 0.82
	Forward			14.82<br>± 0.23	766<br>± 53	0.45<br>± 0.08	5.81<br>± 1.29
0.75M_2kRPM	Reverse	83.0	3	14.67<br>± 0.27	872<br>± 13	0.65<br>± 0.02	9.46<br>± 0.30
	Forward			14.60<br>± 0.29	821<br>± 12	0.56<br>± 0.04	7.69<br>± 0.51
1M_4kRPM	Reverse	88.3	4	14.42<br>± 0.07	851<br>± 37	0.63<br>± 37	8.74<br>± 0.66
	Forward			14.30<br>± 0.10	784<br>± 25	0.53<br>± 0.01	6.79<br>± 0.28
1M_3kRPM	Reverse	97.6	2	14.49<br>± 0.17	865<br>± 5	0.64<br>± 0.02	9.05<br>± 0.52
	Forward			14.35<br>± 0.10	809<br>± 6	0.58<br>± 0.01	7.57<br>± 0.08
1M_2kRPM	Reverse	124.2	4	13.08<br>± 0.26	791<br>± 43	0.62<br>± 0.03	7.29<br>± 0.88
	Forward			13.09<br>± 0.26	749<br>± 49	0.53<br>± 0.02	5.88<br>± 0.68

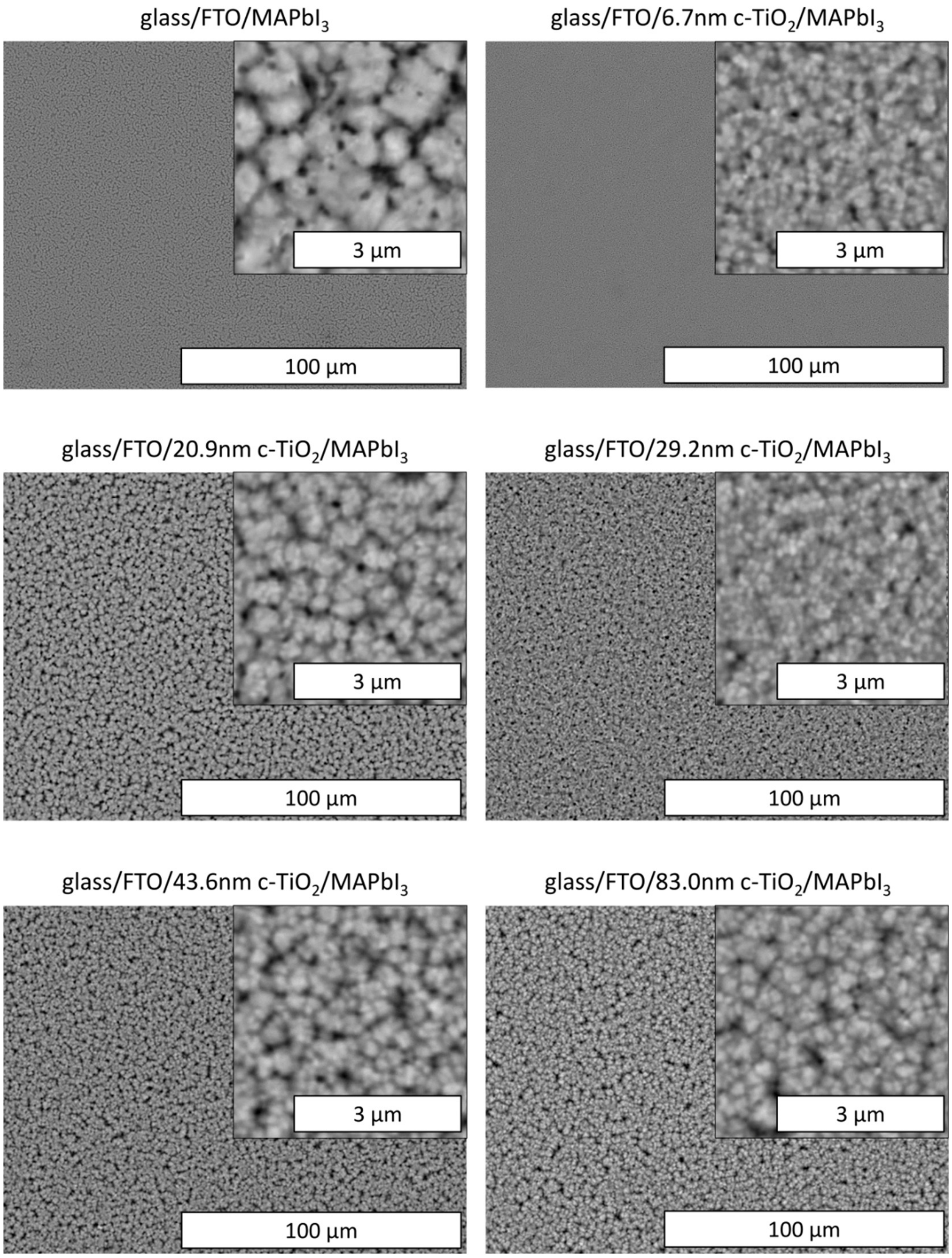

Figure S3. SEM images showing the morphology of the $MAPbI_3$ perovskite layer deposited on glass/FTO/c-TiO_2, where the TiO_2 layer differed in thickness. Scale bars are also presented.
